# Microbial Diversity of Genital Ulcers of HSV-2 Seropositive Women

**DOI:** 10.1038/s41598-017-15554-8

**Published:** 2017-11-13

**Authors:** Supriya D. Mehta, Ashish K. Pradhan, Stefan J. Green, Ankur Naqib, Elijah Odoyo-June, Charlotte A. Gaydos, Sheila Barry, Alan Landay, Robert C. Bailey

**Affiliations:** 1 Division of Epidemiology & Biostatistics, School of Public Health, University of Illinois at Chicago, Chicago, IL USA; 2 Division of Industrial Engineering, School of Engineering, University of Illinois at Chicago, Chicago, IL USA; 3 DNA Services, School of Medicine, University of Illinois at Chicago, Chicago, IL USA; 4 Division of Bioinformatics, Department of Bioengineering, University of Illinois at Chicago, Chicago, IL USA; 5 Centers for Disease Control and Prevention, Kenya; Formerly of Nyanza Reproductive Health Society, Kisumu, Nyanza Province, Kenya; 6 Division of Infectious Diseases, School of Medicine, University of Illinois at Chicago, Chicago, IL USA; 7 Division of Infectious Diseases, Department of Medicine, School of Medicine, Johns Hopkins University, Baltimore, MD USA; 8 Department of Microbiology & Immunology, Rush University, Chicago, IL USA

## Abstract

We measured the microbial community structure of genital ulcers in women. Swabs from clinically detected ulcers were tested for HSV-2 and *Treponema pallidum* by polymerase chain reaction (PCR). HSV-2 and *T*. *pallidum* were detected by serum antibody testing. Microbial community structure was characterized by high-throughput 16 s rRNA gene amplicon sequencing. Multiple group testing and Elastic net and Lasso regressions identified taxa associated with differences in factors of interest. Among 49 ulcer specimens from 49 HSV-2 seropositive women, by PCR HSV-2 was recovered from 28 (57%) specimens and *T*. *pallidum* from none; one woman showed serologic evidence of syphilis. Overall, 63% of women were HIV-positive and 49% had an uncircumcised male sex partner. By both multiple group testing and regression, *Porphyromonas* (FDR p-value = 0.02), *Prevotella* (FDR p-value = 0.03), *Anaerococcus* (FDR p-value = 0.07), and *Dialister* (FDR p-value = 0.09) were detected at higher relative abundance in HSV-2 PCR-positive than negative ulcers. The presence of HSV-2 in a lesion was associated with presumed bacterial agents of Bacterial vaginosis. Differences in bacterial communities may contribute to HSV-2 ulcer pathogenesis, severity, or prolonged healing. If these results are confirmed, future studies may consider the influence of BV treatment on women’s GUD and HSV-2 incidence and recurrence.

## Introduction

Medical male circumcision (MMC) reduces the risk of heterosexual HIV acquisition in African men by ~60%^1–3^. Among Ugandan MMC trial participants, penile anaerobic bacteria were reduced following circumcision^4^. The reduction in penile anaerobic bacteria appears to be transmissible, with a 40–60% reduced risk of bacterial vaginosis (BV) among female sex partners of circumcised men^5^. MMC reduced the risk of genital ulcer disease (GUD) in all three MMC trials by 37–52%^2,3,6^, and reduced GUD by 22% in female sex partners of circumcised men in the Ugandan trial^5^. In our MMC trial in Kenya, despite reduction in GUD, we found no reduction in HSV-2 incidence^6,7^. To identify the cause of GUD reduction, we conducted high-throughput amplicon sequencing of microbial 16 S rRNA genes on clinically detected genital ulcer specimens. *Fusobacteria* and *Sneathia* had nearly 6-fold greater odds of recovery from clinically detected non-herpetic and non-syphilitic GUD compared to STI-associated GUD^8^. These two BV-associated anaerobes are more common among uncircumcised men^4,8^. Bacteria from these genera have inflammatory and tissue destructive properties causing oral mucosal ulcers^9–11^. In light of this novel yet plausible mechanism by which anaerobic bacteria might contribute to genital ulcer pathogenesis, and the relationships between male circumcision status, penile microbiome, GUD, and HIV, we sought to describe the bacterial community structure of GUD in women and whether that differed by ulcer recovery of HSV-2, HIV status, male partner’s circumcision status, and clinical presentation.

## Methods

This study received ethical approval from the Institutional Review Boards of the University of Illinois at Chicago and University of Nairobi. All methods were performed in accordance with relevant guidelines and regulations. Women aged 18 years and older were recruited from sexually transmitted infection (STI) clinic visits where genital ulcers were clinically detected. Consenting women were compensated 200 Kenyan Shillings (~$2.00 USD) for their time. HIV testing was offered to all women and conducted according to Kenyan national guidelines.

### Physical Examination Findings of Genital Ulcer Disease

All patients underwent standardized medical examination and history as part of routine clinical care. External genital and speculum examination by trained clinicians recorded the presence or absence of genital ulcers, the location and number of ulcers. Clinicians swabbed genital ulcers using a Dacron or polyester tipped swab. Women presenting with genital ulcer complaint were told about the study prior to history and examination and if interested, informed consent was obtained at that time. If genital ulcer was detected during the examination, only women who provided informed consent were invited to participate in the study after the completion of the history and examination.

### Socio Demographic and Behavioral Data Collection

Sociodemographic and behavioral risk information obtained by personal interview included age, marital status, educational attainment, employment status, number of circumcised and uncircumcised male sex partners in the last 30 days and last 6 months, male sex partner’s circumcision status at the last sexual intercourse, condom use at last sex and frequency in the past 6 months, and other behaviors expected to be associated with genital microflora exposure.

### Detection of HSV-2 and Treponema pallidum

Serum specimens were tested at the study clinic for HSV-2 antibody (Kalon HSV-2 IgG ELISA, Kalon Biological Limited, Aldershot, United Kingdom) according to the manufacturer’s instructions using recommended cutoffs. Syphilis infection was detected using the rapid plasma reagin test (RPR) (Macro-Vue™, Becton Dickinson, New Jersey, United States), confirmed by the *T*. *pallidum* hemagglutination (TPHA) assay (Randox Laboratories Ltd., Ardmore, United Kingdom).

Genital ulcer specimens were frozen at −20 °C, and shipped to Johns Hopkins University STD Research Laboratory for detection of HSV-1, HSV-2 and *T*. *pallidum* by multiplex polymerase chain reaction (PCR). Swabs were treated with 350 µl of 1X TRIS-EDTA (TE) buffer and vortexed for 15 seconds. The swab was removed from the TE buffer with a pair of tweezers, and the swab was expressed on the side of the tube ~3 times by rotating and pressing it into the tube. The swab was added back to the tube, and 200 µl of the 1X TE buffer was pipetted into a sample processing tray for extraction with the Roche MagNA Pure LC robot, leaving 150 µl for amplicon sequencing. All samples were extracted utilizing the Roche MagNA Pure LC robot with DNA Isolation Kit I, following the DNA I Blood/Cells High Performance extraction protocol and PCR was performed for HSV-1, HSV-2, and *T*. *pallidum* ^12^.

### Analysis of microbial community structure

Genomic DNA (gDNA) from ulcers was PCR amplified and prepared for next-generation sequencing (NGS) using a modified two-step targeted amplicon sequencing approach, similar to that described previously^13,14^, with primers 515 F and 806 R^15^, targeting the V4 variable region of Bacterial and Archaeal small subunit (SSU) ribosomal RNA (rRNA) gene. The primers contained 5′ common sequence tags (common sequence 1 and 2 [CS1 and CS2]) as described previously^16^. The forward primer, CS1–515F (ACACTGACGACATGGTTCTACAGTGCCAGCMGCCGCGGTAA) and reverse primer, CS2-806R (TACGGTAGCAGAGACTTGGTCTGGACTACHVGGGTWTCTAAT) were synthesized by Integrated DNA Technologies (IDT; Coralville, Iowa) as standard oligonucleotides. The PCR amplifications were performed in 10 microliter reactions in 96-well plates. A mastermix for the entire plate was made using the 2X AccuPrime SuperMix II (Life Technologies, Gaithersburg, MD). Final concentration of CS1_515 F and CS2_806 R primers was 500 nM. Cycling conditions were as follows: 95 °C for 5 minutes, followed by 28 cycles of 95 °C for 30”, 55 °C for 45” and 68 °C for 30”. A final, 7 minute elongation step was performed at 68 °C. A single microliter of the first amplification reaction was transferred to a second amplification reaction with the same mastermix conditions, but with different primers. No purification was performed. Each well received a unique primer pair obtained from the Access Array Barcode Library for Illumina sequencers (Fluidigm, South San Francisco, CA; Item# 100–4876), which contain Illumina sequencing adapters, a sample-specific barcode (reverse primer), and CS1 or CS2 sequences. Cycling conditions were as follows: 95 °C for 5 minutes, followed by eight cycles of 95 °C for 30”, 60 °C for 30” and 68 °C for 30”. A final, 7 minute elongation step was performed at 68 °C. Samples were pooled in equal volume and purified using solid phase reversible immobilization (SPRI) cleanup, implemented with AMPure XP beads (Beckman Coulter, Brea, CA, USA) at a ratio of 0.6X (v:v) SPRI solution to sample volume.

The library pool was spiked with 15% non-indexed PhiX control library provided by Illumina and loaded onto a MiSeq v2 flow cell at a concentration of 8 pM for cluster formation and sequencing. Sequencing was performed at the W.M. Keck Center for Comparative and Functional Genomics at the University of Illinois at Urbana-Champaign (UIUC), and data were analyzed using the Casava1.8 pipeline.

Raw sequence data were imported into the software package CLC genomics workbench (v7.0; CLC Bio, Qiagen, Boston, MA). Sequences were merged, quality trimmed (Q20), and filtered to remove residual phiX contamination, and exported as FASTA files. Subsequently, sequence data were processed using the software package QIIME (v1.8.0;^17^). Briefly, sequences were screened for chimeras using the usearch61 algorithm^18^ using *de novo* and reference-based detection methods, and putative chimeric sequences were removed from the dataset. All the reads were then clustered into operational taxonomic units (OTU), using a threshold similarity of 97%. Representative sequences from each OTU were extracted, and these sequences were classified using the “assign_taxonomy” algorithm implementing the RDP classifier, with the Greengenes reference OTU build (v13_8;^19^). A taxon-by-sample abundance matrix (biological observation matrix, BIOM;^20^) was generated at taxonomic levels from phylum to genus using the “make_OTU_table” algorithm. The BIOM was rarefied to 1,250 sequences per sample within QIIME to avoid analytical issues associated with variable sequence number between samples^21^.

### Statistical Analysis

We used three analytic approaches to compare the bacterial community of genital ulcers by detection of HSV-2, male sex partner circumcision status, and HIV status: (1) test for global differences in bacterial communities by factors of interest; (2) compare diversity of bacterial communities by factors of interest; (3) identify specific bacterial taxa differing by factors of interest. Analyses were conducted at the taxonomic level of genus. Additionally, we compared clinical characteristics by recovery of HSV-2 from ulcers. Differences between categorical explanatory variables and factors were assessed by the chi-square test, or Fisher’s exact test when cell size was < 5. Inferential analyses were conducted using STATA/SE 13.0 for Windows (Stata Corp., College Station, TX).

*First*, we tested global bacterial communities by factors using analysis of similarity (ANOSIM). Rarefied sequence data were transformed [log(X + 1)] prior to generation of the Bray-Curtis resemblance matrix. *Second*, diversity at the genus level was measured by the Shannon diversity index (log base *e*) and was compared by factors with Wilcoxon rank sum test. ANOSIM and Shannon diversity index analyses were conducted in the software package Primer6 (Primer-E, version 6.1.13, United Kingdom). *Third*, to identify specific bacterial taxa that differed by HSV-2 PCR result, male partner circumcision status, and HIV status, we compared the number of sequence reads of each genus using the Kruskal-Wallis test. We report test statistic p-values and false discovery rate (FDR)-corrected p-values. Group significance testing was performed within QIIME, employing the Kruskal-Wallis non-parametric test on untransformed number of sequence reads, with calculation of false discovery rate (FDR) corrected p-values^17^. To verify the robustness of these results and considering the dependence between bacterial taxa, we also applied regression to identify specific bacterial bacteria that differed by factors. Elastic net regression was chosen as our primary regression approach as its method of variable selection is not bounded by the number of samples. It uses an additional ridge regression penalty to nullify this constraint^22^. For the validation process, k-fold cross validation was used as the sample size was not sufficient to be divided into sizable training and validation sets. We applied numerous values of k in order to find out the change in the parameters of our equation. We tested numerous values for the ridge regression parameter to identify the optimum model for appropriate variable selection. To assess the robustness of the results of Elastic net, LASSO regression was implemented^23^ and we identified 7 bacteria that were selected by both Elastic net and LASSO regression as having an association with any of the outcomes (HSV-2 status, HIV status, male sex partner circumcision status) at the p < 0.10 level. We applied linear regression to these 7 bacteria for each of the three outcomes, and report coefficients, p-values and adjusted R-squared values, which was multivariable adjusted when a bacterium was found to be significantly associated (p < 0.10) with more than one dependent variable. Before the analysis by linear regression, the data was standardized with µ = 0 and σ = 1. Regression analyses were executed in SAS software, version 9.4 (SAS Institute Inc., Cary, NC, USA).

Principal coordinates analysis was used to visualize bacterial community differences by the three dependent variables (HSV-2 PCR status, HIV status, and male sex partner circumcision status). We calculated both within and between group weighted Unifrac distances to find out the phylogenetic distances between the different groups of samples according to the dependent variables. The Weighted Unifrac distances and principal coordinate analysis plots were operationalized using the *phyloseq*, *ggplot2*, *plyr*, *ape* and *wesanderson* packages in the R 3.3.2 environment (R Development Core Team).

### Data Access

The 16 S rRNA gene amplicon sequence data from this study have been submitted to the NCBI Sequence ReadArchive (http://www.ncbi.nlm.nih.gov/Traces/sra/sra.cgi) under accession number PRJNA281808.

## Results

For this pilot study, the target sample size was 60 subjects, and 58 subjects were enrolled in the allotted timeline. Data were rarified to 1,250 sequences, which resulted in maintaining 56 subjects. Overall, 49 (88%) women were HSV-2 seropositive and 7 (12%) were HSV-2 seronegative. Among the 49 HSV-2 seropositive women, by PCR HSV-2 was detected in 28 (57%) ulcers and *T*. *pallidum* in none. Serologically, syphilis was detected in 1 (2.0%) woman (titer 1:4; TPHA-positive); there were no sequence reads from the genus *Treponema* detected in this specimen (0/34,176 sequences before rarifaction). One serologically negative subject had an OTU annotated as *Treponema* (30/47,102 sequences before rarefaction) with HSV-2 recovered from the ulcer. Among the 7 HSV-2 seronegative women, there was no serologic evidence of syphilis. HSV-2 was PCR-detected in three (38%) of seven ulcers from HSV-2 seronegative women while *T*. *pallidum* was not detected; amplicon sequencing did not identify any *Treponema* spp. sequence reads in HSV-2 seronegative women. All GUD specimens were negative for HSV-1. Therefore, no STI etiology was identified in four (7%) of 56 women with clinically detected genital ulcers. Given the small number of HSV-2 seronegative subjects, inferential analyses were restricted to the 49 HSV-2 seropositive women. From these 49 specimens we obtained 1,664,532 sequences with an average read-length of 250 base pairs. An average of 33,970 sequences were obtained from each sample (min = 1,261, max = 64,939).

### Socio-Demographic and Behavioral Characteristics

HSV-2 seropositive women were median age 26 years (range 18–59) and 76% married or cohabiting (Table 1). Overall, 60% of women reported having one sex partner in the past six months, with 17% reporting two or more sex partners, and 49% reported having any uncircumcised sex partner in the past six months. Male sex partner’s circumcision status at the last sex, past 30 days, and past six months were highly correlated (*Pearson correlation coefficient* = 0.68–0.79); subsequent analyses proceeded with circumcision status in the past six months as most women reported one sex partner in the past six months. Having an uncircumcised male sex partner did not differ between women with HSV-2 PCR positive (50%) or negative (47%) ulcers (p = 0.86). Overall, 63% of women were HIV positive, and this did not differ significantly between women with HSV-2 PCR positive or negative ulcers (71% vs. 50%, p = 0.13). There were no statistically significant differences in sociodemographic, behavioral characteristics, or the joint distribution of HIV status and male sex partner circumcision status between women with HSV-2 PCR positive or negative ulcers (Table 1).

**Table 1:** Subject Characteristics by HSV-2 mPCR Results.

Characteristic*	HSV-2 mPCR Results		P-Value
	Positive, N = 28 n (%)	Negative, N = 21 n (%)	
Median age in years	26.5	26	0.87
HIV status			
Negative	8 (29)	10 (50)	0.13
Positive	20 (71)	10 (50)	
Antiretroviral use reported	8 (40)	5 (50)	
Married or cohabiting			
No	8 (29)	4 (19)	0.52
Yes	20 (71)	17 (81)	
Educational attainment			
Primary or less	17 (61)	16 (76)	0.25
Some secondary or higher	11 (39)	5 (24)	
Used antibiotics, past 1 month			
No	12 (43)	8 (38)	0.74
Yes	16 (57)	13 (62)	
^Uncircumcised male sex partner last 6 months			
No	14 (50)	10 (53)	0.86
Yes	14 (50)	9 (47)	
HIV status and male partner circumcision status			
HIV negative, circumcised partner	6 (33)	3 (11)	0.25
HIV positive, circumcised partner	3 (17)	10 (37)	
HIV negative, uncircumcised partner	3 (17)	4 (15)	
HIV positive, uncircumcised partner	6 (33)	10 (37)	
Number of sex partners			
None	8 (29)	3 (15)	0.55
One	15 (53)	14 (70)	
Two or more	5 (18)	3 (15)	
Condom used at last sex			
No	12 (43)	14 (67)	0.1
Yes	16 (57)	7 (33)	
Median Shannon diversity index [95% CI]	2.1 [1.8–2.3]	1.9 [1.3–2.1]	0.05
Genus-level, log base *e*			
Median number of genus-level bacterial taxa [95% CI]	26.5 [22–35]	17 [13–23]	0.02

^Uncircumcised represents having any uncircumcised sex partner in the past 6 months; Circumcised represents all sex partners in the past 6 months were circumcised.

*Not all cells sum to N due to missing responses. ^+^Recall period for sexual behaviors is past 6 months, unless otherwise specified.

CI = Confidence Interval.

### Clinical Characteristics by HSV-2 PCR Results

Compared to HSV-2 positive ulcers, the treating clinician was more likely to describe HSV-2 PCR negative ulcers as papular (32% vs. 4%, p = 0.02), and less likely to describe HSV-2 PCR negative ulcers as “patch” (0% vs. 21%, p = 0.03), and hyperpigmented (18% vs. 47%, p = 0.08; Table 2). The clinical impression was associated with PCR detection of HSV-2: 54% of ulcers in which HSV-2 was recovered were diagnosed as “GUD of herpetic appearance” compared to 24% of those that were HSV-2 negative (p = 0.05).

**Table 2:** Clinical Characteristics by HSV-2 mPCR Results.

Characteristic*	HSV-2 mPCR Results		P-value
	Positive, N = 28	Negative, N = 21	
	n (%)	n (%)	
Symptoms^			
Vaginal discharge (n = 48)	10 (37)	10 (48)	0.46
Genital itching	13 (46)	12 (57)	0.46
Painful urination	17 (61)	10 (48)	0.36
Lower abdominal pain (n = 48)	4 (15)	5 (24)	0.48
Number of ulcers			
1	14 (50)	9 (43)	
2–3	11 (39)	5 (24)	
4–10	3 (11)	7 (33)	0.14
Median duration of ulcer (IQR) (n = 43)	7 (4–25.5)	6.5 (4–10.5)	0.24
Ulcer location^			
Labia majora	23 (82)	15 (71)	0.37
Labia minora	5 (18)	4 (19)	1
Introitus	3 (11)	2 (10)	1
Vault	2 (7)	2 (10)	1
Ulcer description^			
Multiple (vs. Singular)	15 (54)	12 (57)	0.8
Macular (n = 47)	22 (79)	11 (58)	0.13
Papular (n = 46)	1 (4)	6 (32)	0.02
Pustular (n = 46)	0 (0)	2 (11)	0.17
Patch (n = 48)	6 (21)	0 (0)	0.03
Hyperpigmentation (n = 36)	9 (47)	3 (18)	0.08
Hypopigmentation (n = 36)	5 (26)	8 (47)	0.2
Other Examination findings^			
Discharge from os (n = 45)	10 (40)	7 (35)	0.73
Cervical friability/bleeding (n = 45)	2 (8)	1 (5)	1
Clinical impression^			
GUD, herpetic	15 (54)	5 (25)	0.05
GUD, non-herpetic	15 (54)	15 (75)	0.13
Vaginitis	3 (11)	6 (32)	0.13
Medications dispensed^+^			
Acyclovir only	5 (18)	5 (24)	
Acyclovir, penicillin, erythromycin	10 (36)	3 (14)	
Penicillin, erythromycin	7 (25)	12 (57)	
Erythromycin only	3 (11)	1 (5)	
Penicillin only	2 (7)	0 (0)	
Acyclovir, erythromycin	1 (4)	0 (0)	0.13

^*^Not all cells sum to N due to missing data; the sample size for variables with missing responses are indicated.

^Not mutually exclusive.

^+^Acyclovir dispensed as cream or tabs; Penicillin dispensed as Penicillin G 2.4 million units.

IQR = Interquartile range.

### Global Comparison of Microbial Community Structure by mPCR Results

**Figure 1:**
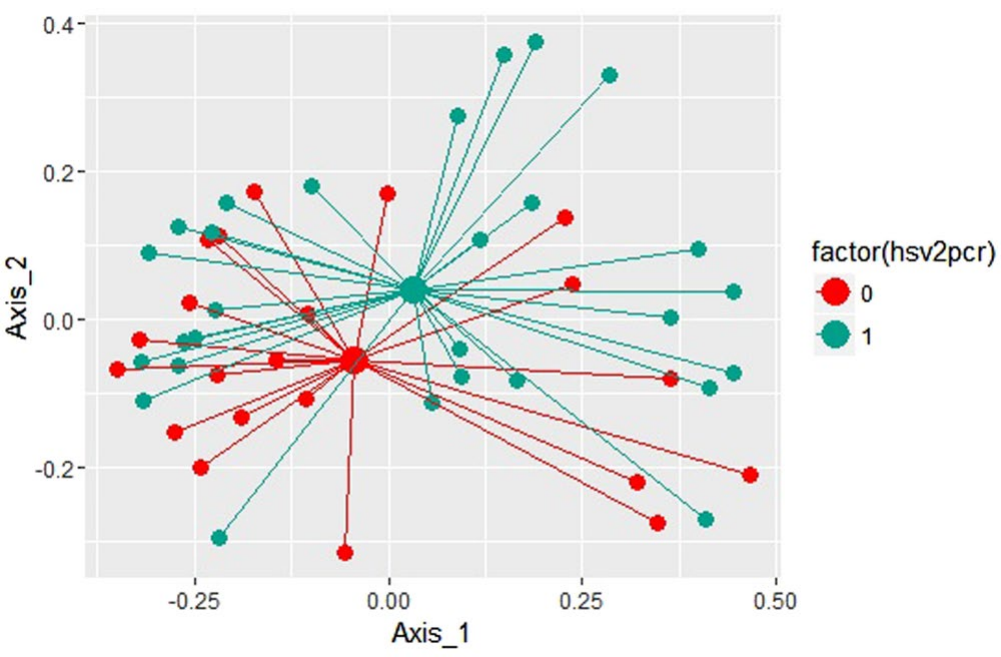
Principal coordinates analysis of Weighted UniFrac values between HSV-2 PCR positive (blue) and HSV-2 PCR negative (red) samples with the axes scaled by the percentage of the variance that they contain to summarize the microbial community compositional differences between samples. Each point represents a single sample, and the distance between points represents how compositionally different the samples are from one another.

**Figure 2:**
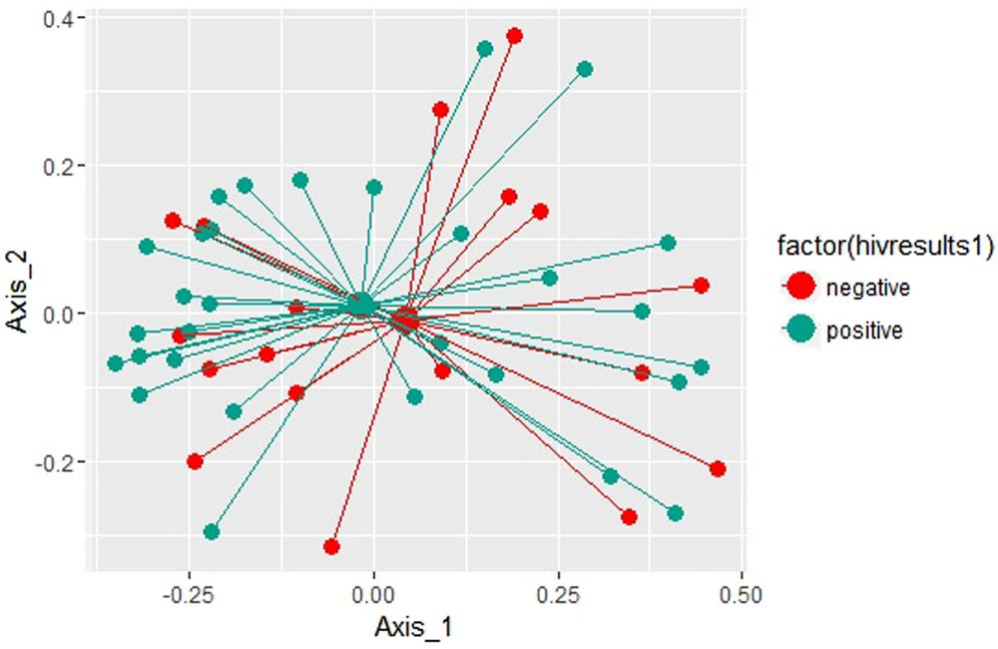
Principal coordinates analysis of Weighted UniFrac values between HIV positive positive (blue) and HIV negative (red) samples.

**Figure 3:**
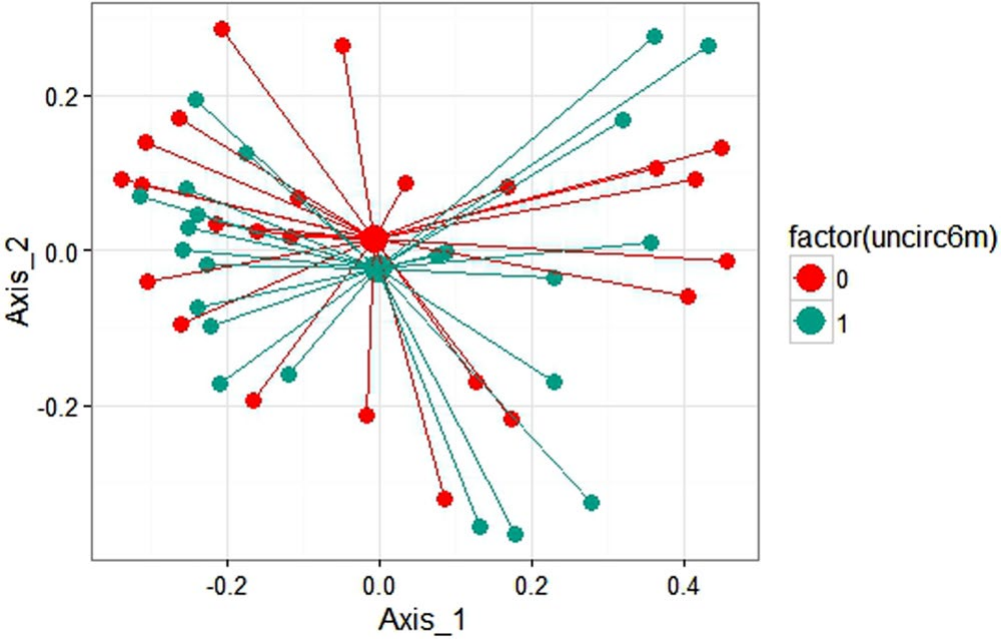
Principal coordinates analysis of Weighted UniFrac values between samples from women with uncircumcised male sex partners (blue) and circumcised male sex partners (red).

Analysis of similarity (Table 3) showed that the difference in bacterial community composition between HSV-2 PCR-positive and PCR-negative women was statistically significant (p = 0.002). There were no statistically significant differences in community composition by HIV status or male sex partner circumcision status. The principal components plots are complementary visualization to these analytic results: the community centroid for samples from women with HSV-2 PCR positive ulcers are distinct from the centroid of samples from women whose ulcers were HSV-2 PCR negative (Fig. 1), while centroids representing bacterial communities by HIV status (Fig. 2) and male sex partner circumcision status (Fig. 3) show much greater overlap and shorter distance between centroid.

**Table 3:** Results of Analysis of Similarity Measures: Comparison of Microbial Community Structure by Patient Factors.

Variable	Global R statistic	P-value
*All samples* (N = 49)		
HSV-2 PCR results	0.148	0.002
Circumcision status of male sex partner	0.010	0.277
HIV status	0.011	0.315
*HSV-2 PCR Positive* (N = 28)		
Circumcision status of male sex partner	−0.028	0.766
HIV status	−0.050	0.693
*HSV-2 PCR Negative* (N = 21)		
Circumcision status of male sex partner	0.165	0.053
HIV status	0.273	0.011

As reflected by the median Shannon index (Table 1), there was greater diversity in microbial communities from ulcers in which HSV-2 was detected compared to ulcers in which HSV-2 was not detected (1.9 vs. 2.1, p = 0.05). The median number of taxa recovered was also higher for ulcers in which HSV-2 was detected (26.5 vs. 17, p = 0.02).

### Specific Bacterial Taxa Differing by HSV-2 Status, HIV Status, and Male Sex Partner Circumcision Status

In Kruskal-Wallis analysis, compared to HSV-2 PCR negative ulcers (Table 4), HSV-2 PCR positive ulcers had higher mean sequence count for bacteria from the genera *Porphyromonas* (FDR p = 0.02) and *Prevotella* (FDR p = 0.03), with strong trends for *Anaerococcus* (FDR p = 0.07) and *Dialister* (FDR p = 0.09) (Table 3). While *Finegoldia*, *Gardnerella*, *Corynebacterium*, and *Anaerococcus* demonstrated higher mean sequence count for ulcers from HIV-positive women compared to ulcers from HIV-negative women (p < 0.05 each), only the difference for *Finegoldia* trended toward FDR significance (FDR p = 0.09). This was echoed in regression analyses, with a statistically significant 0.81 standard deviation reduction in *Finegoldia* for HIV-negative women (Table 5). Only the mean sequence of *Gardnerella* differed by male sex partner circumcision status (p = 0.01), and the FDR p-value was non-significant (p = 0.20). Results of regression (Table 5) also identified *Porphyromonas* as a significant predictor of HSV-2 positivity and *Finegoldia* with HIV positivity.

**Table 4:** Mean sequence reads of bacterial taxa by HSV-2 PCR status, HIV status, and male sex partner circumcision status with Kruskall-Wallis group tests of significance

Bacteria	HSV-2 PCR status		P-value	FDR P-value	HIV Status		P-value	FDR P-value	Male partner circumcision status		P-value	FDR P-value
	Positive	Negative			Positive	Negative			Uncircum	Circum		
*Porphyromonas*	22	1	<0.01	0.02	13	13	0.79	0.79	10	17	0.72	0.95
*Prevotella*	156	80	<0.01	0.03	145	95	0.85	0.90	141	113	0.09	0.41
*Anaerococcus*	111	52	0.01	0.07	81	99	0.04	0.15	90	88	0.87	0.94
*Dialister*	18	18	0.02	0.09	15	24	0.79	0.90	13	24	0.37	0.72
*Sneathia*	100	22	0.03	0.11	93	27	0.10	0.29	112	29	0.12	0.41
*Finegoldia*	62	46	0.17	0.44	33	95	0.01	0.09	51	63	0.83	0.94
*Peptoniphilus*	93	93	0.25	0.57	84	113	0.48	0.59	123	73	0.45	0.72
*Acinetobacter*	27	87	0.41	0.81	46	66	0.11	0.29	27	65	0.13	0.41
*Corynebacterium*	41	44	0.53	0.88	24	75	0.03	0.15	45	43	0.97	0.97
*Staphylococcus*	19	101	0.56	0.88	21	111	0.15	0.29	13	88	0.11	0.41
*Gardnerella*	88	95	0.70	0.88	125	41	0.01	0.11	125	50	0.01	0.20
*Lactobacillus*	165	254	0.75	0.88	176	190	0.32	0.51	164	226	0.50	0.72
*Streptococcus*	21	21	0.77	0.88	13	58	0.14	0.29	35	26	0.47	0.72
*Aerococcus*	2	13	0.96	0.96	11	0	0.15	0.40	14	1	0.29	0.77

FDR P-value = False discovery rate corrected P-value

“Uncircum” = Uncircumcised; “Circum” = Circumcised.

We present results from the 14 bacteria representing the top 10 bacteria of significance (ranked by P-value) for each factor. Because some bacteria were in the top 10 for some outcomes but not others (e.g., *Gardnerella* for HIV status and male sex partner circumcision status, but not for HSV-2 status), this leads to presentation of more than 10 bacteria total.

**Table 5:** Results of linear regression: Bacteria associated with HSV-2, HIV, and circumcision status.

Genus	HSV-2 positivity	HIV positivity	Uncircumcised male partner	P-value	R-squared Adjusted
*Porphyromonas*	0.91 (0.26)	—	—	0.0010	0.1901
*Peptostreptococcus*	0.83 (0.27)	—	—	0.0029	0.1556
*Adlercreutzia*	0.80 (0.27)	—	—	0.0043	0.1430
*Finegoldia*	—	−0.81 (0.28)	—	0.0049	0.1411
*WAL_1855D*	0.70 (0.27)	—	—	0.0135	0.1044
*Sneathia*	0.66 (0.28)	—	0.44 (0.28)	0.0237	0.1180
*Gardnerella*	—	—	0.59 (0.28)	0.0391	0.0710
*Phascolarctobacterium*	—	—	0.53 (0.29)	0.0699	0.0505

Interpretation: These results represent 7 different models for the 7 bacteria identified in Elasticnet and Lasso regression as associated with HSV-2 ulcer status, HIV status, and/or male sex partner’s circumcision status at the p < 0.10 level. As *Sneathia* was associated with both HSV-2 status and male sex partner circumcision status, the model controlled for both factors. Data are standardized (µ = 0 and σ = 1) and we report the coefficient (standard error), model p-value and adjusted R-squared.

## Discussion

Among HSV-2 seropositive women, HSV-2 was detected by PCR in 57% of clinically identified ulcers, and the bacterial community of these ulcers differed by whether HSV-2 was detected with PCR. All women were HSV-2 seropositive; the extent to which it matters that GUD of putatively similar etiology has variation in bacterial community composition relates to the potential association between these different bacterial community structures with co-infections or impact on severity of disease or treatment.

This study was conducted as response to our previous finding that 55% of men with clinically detected genital ulcers were serologically negative for HSV-2 and syphilis, and 39% also had no HSV-2 or *T*. *pallidum* recovered by PCR^8^. This high proportion of non-etiologically defined GUD is in keeping with studies throughout Sub-Saharan Africa^24–29^ and India^30,31^. Using amplicon sequencing in our study of men, we found that anaerobic bacteria associated with BV were more commonly recovered from genital ulcers in these men without HSV-2 or *T*. *pallidum*. Our current findings present two striking differences to these previous results. First, a minority of women (9%) with GUD were without STI-associated etiology. Subjects in the current study self-presented with symptoms, whereas in the previous study, samples were collected from men taking part in a randomized clinical trial of MMC to reduce HIV incidence^3^. Men in the trial underwent physical examination every 6 months, and were encouraged to come to the clinic for symptoms at interim visits. HIV was rare (4%) in our prior analysis as men had to be HIV negative for trial entry, compared to 63% HIV-positivity among women in the current study. HIV can alter the natural history of genital herpes, producing more severe and frequent outbreaks and delayed healing^32^. Therefore, in the trial we increased our chance of detecting non-STI related ulcers and ulcers that may not have been severe enough to lead to health care seeking otherwise. Secondly, our present study found that ulcers in which HSV-2 was detected by PCR had *more* BV-associated bacteria – specifically, *Porphyromonas*, *Prevotella*, *Peptostreptococcus*, *Sneathia*, and possibly *Anaerococcus* and *Dialister –* than ulcers from which HSV-2 was not detected. Ulcers in which HSV-2 was not detected by PCR may have been at a different point in healing, and therefore HSV-2 was no longer detectable. However, the median duration of ulcer symptoms did not differ by whether or not HSV-2 was recovered from the ulcer. In a cohort of Kenyan women, Kaul *et al*. found that baseline HSV-2 infection was associated with increased incidence of BV^33^. At the same time, prospective epidemiologic and clinical investigations support the increased risk of HSV-2 acquisition among women with BV^34^. Whether HSV-2 increases risk of BV and/or BV increases risk of HSV-2, such association would increase the likelihood of co-detection of HSV-2 and BV-associated bacteria in cross-sectional study design. Though published data regarding the effect of BV on the natural history of HSV-2 are limited, Ursell *et al*. demonstrate that BV-associated bacteria (*G*. *vaginalis*, *A*. *vaginae*) have been associated with increased duration and number of HSV-2 related outbreaks^35^.

A primary public health concern is that among HSV-2 seropositive individuals there is increased risk of HIV acquisition^36^ and transmission^37^, highlighted by the 63% HIV prevalence among our sample of HSV-2 infected women. We detected significant global bacterial community dissimilarity between HIV-positive and HIV-negative women only among ulcers that were HSV-2 PCR negative. We observed that *Finegoldia* was significantly reduced among women with HIV. Prospective studies of the effect of HIV on the vaginal microbiome are limited, but our recent analysis of a cohort of U.S. women showed that HIV-status did not alter the change in the vaginal microbiome over an 8- to 10-year period^38^.

The microbial community of ulcers from women with an uncircumcised partner reflected more BV-associated bacteria. This is consistent with what we expected based on our previous findings and those of others showing that female partners of uncircumcised men are more likely to have BV, and that this stems from the greater abundance of BV-associated anaerobic bacteria among uncircumcised men compared to circumcised men^39^. However, in our study this difference was only observed in ulcers from which HSV-2 was not recovered. We are unable to determine why the differences by HIV status and male partner circumcision status were detected only among HSV-2 PCR negative ulcers. Although these women were all HSV-2 seropositive, the GUD detected could have had different or multiple etiologic factors. This may be supported by findings of the differences in ulcer characteristics between HSV-2 PCR positive and negative ulcers, and overall, the clinical impression was less often “GUD, herpetic” for PCR negative (25%) than positive ulcers (54%).

### Limitations

We recruited women seeking care at an STI clinic, which likely biased our sample towards those with more severe and HSV-2 related GUD. Data on male partner circumcision status were self-reported by women, and there may have been misclassification. In a study of women’s knowledge of partners’ male circumcision status in Uganda, 1.2% of women misreported the status of confirmed circumcised men and 8.2% of women misreported the status of uncircumcised men^40^, though study of this in Zambia and Swaziland find misclassification rates 11–15% for either status^41^. We did not conduct species-level analysis, which will be necessary in subsequent studies with more specific hypotheses. This pilot study had low power to detect modest associations for individual bacteria, especially in the context of multiple testing correction, which was necessary for hypothesis exploration. However, we used multiple analytic approaches to increase confidence in the robustness of results: detection of differences is supported by results of ANOSIM, Bray-Curtis dissimilarity measure, Kruskal-Wallis group testing, and regression analyses, with convergence on specific bacteria. We did not collect data on frequency of previous GUD, occurrence of prodrome, or clinician characterized stage of healing. We did not diagnose BV by Amsel’s criteria or Nugent score, as the clinic at which the study took place provides only syndromic diagnosis. This information would have aided interpretation of the observed differences in bacterial community structure. These data emphasize the need for longitudinal study to understand the relationships between the vaginal microbiome and women’s risk of HSV-2, HIV, BV, and the role of male sex partner’s circumcision status in these outcomes.

## Conclusions

Among HSV-2 seropositive women, the bacterial community of clinically detected GUD was more diverse and had higher abundance of BV-associated bacteria when HSV-2 was detected in the ulcer. This is of public health and clinical importance as differences in bacterial communities may contribute to HSV-2 related lesion induction, ulcer pathogenesis, severity, or prolonged healing. If these results are confirmed, future studies may consider the influence of BV treatment on women’s GUD and HSV-2 incidence and recurrence.
